# Effects of Physical Activity on Ageism and Aging Anxiety Among Chinese and Korean Adults Aged 55 to 64 Years

**DOI:** 10.3390/healthcare13111218

**Published:** 2025-05-22

**Authors:** Jing Li, Seung-Yong Kim, Cho-Young Yook, Xiao-Long Chen, Woo-Jin An, Ju-Young Oh, Chae-Hee Park

**Affiliations:** 1 Department of Physical Education, Xianyang Normal University, Xianyang 712000, China; lijing@xync.edu.cn (J.L.); chenxl@xync.edu.cn (X.-L.C.); 2 Department of Leisure Sports, Dong Seoul University, Seongnam-si 13117, Republic of Korea; 3 Department of Sport and Healthy, Korea National Sport University, Seoul 05541, Republic of Korea; 63yook@knsu.ac.kr; 4 Department of Sport and Healthy Aging, Korea National Sport University, Seoul 05541, Republic of Korea; 210100@knsu.ac.kr (W.-J.A.); 20230074@m365.knsu.ac.kr (J.-Y.O.)

**Keywords:** ageism, aging, anxiety, physical activity, pre-elderly

## Abstract

**Background**: China and the Republic of Korea, two countries facing rapid population aging, are actively promoting healthy aging. Physical activity (PA), aging, anxiety, depression, and discrimination determine the health and quality of life of older adults. This study compared the levels of PA, ageism, and aging anxiety among Chinese and Korean adults aged 55–64 years. In this study, we explored the effect of PA on ageism and aging anxiety in these individuals. **Methods***:* We surveyed 200 pre-elderly individuals in Shaanxi Province, China, and 201 pre-elderly individuals in Gyeonggi-do, Republic of Korea. The survey assessed PA, ageism, and aging anxiety levels and collected data on nationality, sex, body mass index, marital status, smoking status, and education level. The collected data were analyzed using exploratory and confirmatory factor analyses, *t*-tests, analysis of variance, and structural equation model path analysis, which were performed using SPSS and AMOS. **Results**: The PA level was higher among the Korean participants than among the Chinese participants (*p* = 0.027). In contrast, ageism (*p* < 0.001) and aging anxiety (*p* = 0.001) levels were higher among Chinese participants than among Korean participants. PA was negatively related to aging anxiety (*p* = 0.044) but did not affect ageism (*p* > 0.05). Furthermore, ageism was positively correlated with aging anxiety (*p* = 0.002). **Conclusions**: Pre-elderly Chinese and Korean individuals should be aware of the importance of PA and increase its presence in daily life. Pre-elderly individuals must be prepared for healthy aging. This can be achieved by implementing social policies and increasing awareness of healthy aging.

## 1. Introduction

Population aging is rapidly increasing worldwide. According to the United Nations, the number of people aged 60 years and above is expected to increase from 1 billion in 2019 to 2.1 billion by 2050 [1]. Currently, the number of individuals aged 60 years and above is the highest in China, and by 2025, this number is expected to exceed 300 million. Simultaneously, China’s second baby boomer generation (individuals born between 1962 and 1975) will enter old age, contributing to a substantial demographic shift in the country’s aging population. China is expected to become a super-aged society by 2033, with the proportion of older adults increasing to 29% by 2050 [2]. A super-aged society is one in which at least 20% of the population is aged 65 years or older (World Health Organization [3] and Organization for Economic Co-operation and Development [4]). The Republic of Korea has already transitioned to a super-aged society [5].

All countries, including China and the Republic of Korea, actively promote healthy aging. The World Health Organization defines healthy aging as the process of developing and maintaining functional abilities to maintain good health in older age [6]. This helps to slow the aging process and promotes both the individual and social development of older adults. Age-related declines in physical function and quality of life, particularly decreased aerobic capacity, muscle function, and postural balance, impede activities of daily living and increase disease burden and health costs [7]. Therefore, maintaining physical function has become a public health need, and many health organizations recognize the role of physical activity (PA) in disease prevention and the maintenance of functional independence.

Insufficient PA is one of the leading causes of morbidity and mortality worldwide [8]. According to the Global Health Report, one in four adults (27.5%) and more than three-quarters (81%) of adolescents do not meet the World Health Organization’s recommendations for aerobic PA [9,10]. Performing regular PA is an effective way of improving health, especially among older adults, who tend to experience chronic diseases [11], along with anxiety, depression, and discrimination. Anxiety, the most prevalent psychiatric disorder, is associated with a high disease burden and is often underrecognized and undertreated in primary care [12]. The prevalence of anxiety disorders is as high as 15% in community samples and 28% in clinical samples of older adults [13].

PA has been described as a miracle drug [14] due to its positive effects on physical and mental health; it has been recommended as one of the non-pharmacological methods of reducing anxiety, which is common in older adults [15], and is suitable for everyone [16]. Thus, older adults should be as physically active as their functional ability allows [6]. Age discrimination is the fourth most prevalent form of social discrimination after racial, sex, and gender discrimination. Although age discrimination can affect people of any age, older adults are reportedly at a higher risk of experiencing this discrimination [17], which is a major obstacle to achieving healthy aging [18].

The experiences and health of older adults vary widely. When these differences are overlooked, we risk overgeneralizing and stereotyping older adults, aging, and the concept of old age. This stereotyping of older adults, aging, and old age is known as ageism. Ageism is pervasive and evident in our perceptions of older adults and our interactions with them [19]. Often, we are unaware of our ageist beliefs and behaviors [20] and may even view ourselves from an ageist perspective as older adults. To study ageism in different societies, one must examine age-specific practices and their impact on various groups within society rather than focusing solely on a single age group [21]. This study focused on middle-aged and older individuals approaching later stages of life. Although middle-aged people may consider themselves distinct from older people, this distinction is not fixed. Age groups are not static categories; this is reflected in the fact that older people were once young individuals, while young people, if they survive and stay healthy, will one day become older.

**Figure 1:**
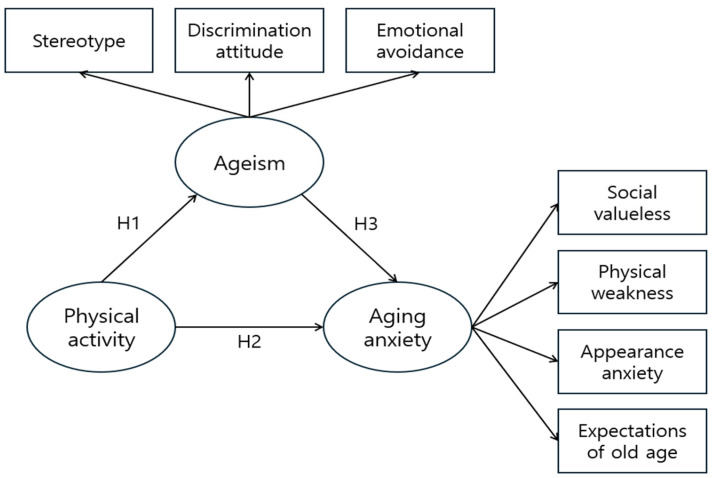
Research model.

This study compared the levels of PA, ageism, and aging anxiety among pre-elderly individuals (aged 55–64 years) in China and the Republic of Korea. This study investigated the effect of physical exercise on ageism and aging anxiety among pre-elderly individuals. To determine the relationship between PA, ageism, and aging anxiety, we hypothesized that PA has a significant negative impact on both ageism (H1) and aging anxiety (H2), while ageism has a significant positive impact on aging anxiety (H3). By examining the relationship between PA, ageism, and aging anxiety, we aimed to help middle-aged and older individuals improve their psychosocial well-being as they approach old age, thus fostering a positive and healthy lifestyle later in life. The research model is illustrated in Figure 1.

## 2. Materials and Methods

### 2.1. Participants

In this study, we included individuals aged 55–64 years living in Shaanxi Province, China, and Gyeonggi-do, Seoul, Republic of Korea. Participants were recruited using a stratified sampling methodology. Data were collected through surveys conducted via online and on-site questionnaires that were distributed and collected. Participants with mental illness and dementia were excluded from the survey. All participants received a modest thank-you gift in accordance with the ethical guidelines.

The sample size was calculated using G* Power software (G* Power 3.1.7, Heinrich-Heine-University, Düsseldorf, Germany). For an effect size of 0.4, α level of 0.05, and test power of 0.9, the minimum sample size was 164 for each group. We conducted stratified sampling based on nationality, sex, age, body mass index (BMI; calculated as weight/[height]^2^), marital status, smoking status, and educational level. A total of 526 questionnaires were distributed, and 200 and 201 valid questionnaires were collected from China and the Republic of Korea, respectively. This study was approved by the Institutional Review Board of the Korea National Sport University (1263-202306-HR-064-01) and was conducted in accordance with the guidelines of the approving body and of the Declaration of Helsinki. Importantly, the Korea National Sport University IRB approval allowed for data collection from both Korea and China. Table 1 presents the general characteristics of the participants.

### 2.2. Measurement of PA

PA was measured using the short version of the International Physical Activity Questionnaire (IPAQ) [22]. The validated Korean version of this questionnaire [23] was used for Korean participants, whereas the validated Chinese version [24] was used for Chinese participants. In this study, the daily activities of middle-aged and older participants were measured according to standards established by the IPAQ. The total amount of PA was calculated, and the intensity was determined as described by Craig et al. [25]. The metabolic equivalent of task (MET) is an essential indicator of exercise intensity, reflecting the level of human metabolism and energy expenditure. One MET is equivalent to approximately 3.0 mL/kg/min oxygen consumption. The metabolic rate of a body is 1 MET at rest, 3.3 MET for walking, 4.0 MET for moderate PA, and 8.0 MET for vigorous PA.

### 2.3. Measurement of Ageism

Ageism was measured using a questionnaire developed based on Fraboni et al.’s questionnaire [26] and modified according to Kim et al. [27]. The questionnaire comprised 18 questions across three factors: emotional avoidance, discrimination attitude, and stereotypes. The Cronbach α was 0.795, 0.796, and 0.794 for emotional avoidance, discrimination attitude, and stereotypes, respectively.

### 2.4. Measurement of Aging Anxiety

Aging anxiety was measured using a questionnaire developed by Lee and You [28] based on a questionnaire developed by Lasher and Fauikender [29]. The questionnaire comprised 19 questions across four factors: physical weakness, appearance anxiety, social worthlessness, and expectations of old age. The Cronbach α was 0.832, 0.817, 0.903, and 0.807 for physical weakness, appearance anxiety, social worthlessness, and expectations of old age, respectively.

### 2.5. Data Analysis

We conducted exploratory and confirmatory factor analyses to test the reliability and validity of the scales used in this study. We also verified whether the average variance extracted (AVE) and composite reliability (CR) exceeded 0.500 and 0.700, respectively [30,31]. Next, we compared the levels of PA, ageism, and aging anxiety based on nationality, sex, body mass index, marital status, smoking status, and education level using t-tests and analysis of variance. Before verifying the hypotheses, we examined the suitability of the research model for empirical analysis. Subsequently, we verified the hypotheses using structural equation modeling path analysis. All statistical analyses were performed using SPSS and AMOS software (version 25.0, IBM Corp., Armonk, NY, USA). Statistical significance was set at *p* < 0.05.

## 3. Results

### 3.1. Reliability and Validity of Employed Scales 

All scales represented 5-point Likert scales ranging from 1 (“strongly disagree”) to 5 (“strongly agree”). Table 2 presents the results of the reliability and validity tests of the scales employed in this study. The Cronbach α was above 0.800 for all variables, indicating that the internal consistency of the latent variable was high and its reliability was good. Meanwhile, the AVE and CR of the model exceeded 0.500 and 0.700, respectively, indicating that the research model had good aggregation validity.

### 3.2. Comparison of PA, Ageism, and Aging Anxiety Based on Nationality, Sex, Body Mass Index, Marital Status, Smoking Status, and Education Level

Table 3 presents the results of comparing the levels of PA, ageism, and aging anxiety among the participants based on nationality, sex, body mass index, marital status, smoking status, and education level. PA (*p* = 0.027), ageism (*p* < 0.001), and aging anxiety (*p* = 0.001) differed significantly by nationality. The PA level was higher among the Korean participants (M = 3155.52) than among the Chinese participants (M = 2541.73). In contrast, the levels of ageism and aging anxiety were higher among the Chinese participants (ageism M = 3.04, aging anxiety M = 3.16) than among the Korean participants (ageism M = 2.74, aging anxiety M = 2.97).

### 3.3. Suitability of the Research Model

We established a structural equation model to explore the impact of PA on ageism and aging anxiety among pre-elderly individuals in China and the Republic of Korea. This research model fit well, with the goodness-of-fit index, incremental fit index, Tucker–Lewis index, and comparative fit index exceeding 0.900, and the root mean square error of approximation was less than 0.100 (Table 4). All fit indices were within a reasonable range, meeting the test criteria for moderation [32]. Thus, we concluded that the theoretical model aligns well with survey data and is suitable for empirical analysis.

### 3.4. Verification of Hypotheses

Table 5 presents the results of the analysis of the path relationships between PA, ageism, and aging anxiety. The results for H1 showed that PA did not affect emotional avoidance (β = −0.058, *p* = 0.298; *p* > 0.05), discrimination attitude (β = −0.061, *p* = 0.118; *p* > 0.05), or stereotypes (β = −0.011, *p* = 0.830; *p* > 0.05), indicating that the amount of PA did not affect age discrimination among pre-elderly individuals.

The results for H2 showed that PA had a significantly negative effect on the physical weakness aspect of aging anxiety (β = −0.164, *p* = 0.044; *p* < 0.05). This result suggests that as the amount of PA increases, the rate of physical weakness decreases among pre-elderly individuals. However, PA did not affect other aspects of aging anxiety, such as social worthlessness, appearance anxiety, or expectations of old age.

The results for H3 showed that emotional avoidance did not affect aging anxiety. However, attitude toward discrimination had a significantly positive effect on the social worthlessness (β = 0.260, *p* < 0.001) and appearance anxiety (β = 0.182, *p* = 0.002) aspects of aging anxiety. This result indicates that the more serious the differential treatment of pre-elderly individuals, the stronger their sense of social worthlessness and the more worried they become about changes in their appearance during the aging process. Furthermore, differential treatment had a significantly negative effect on participants’ expectations of old age (β = −0.165, *p* = 0.034; *p* < 0.05). This result suggests that the more severe the differential treatment of pre-elderly individuals, the more negative their expectations of old age. The stereotyping aspect of ageism had a significantly positive impact on the social worthlessness (β = 0.448, *p* < 0.001; *p* < 0.001), physical weakness (β = 0.527, *p* < 0.001; *p* < 0.001), and appearance anxiety (β = 0.459, *p* < 0.001; *p* < 0.001) aspects of aging anxiety.

## 4. Discussion

In this study, we investigated the effects of physical exercise on ageism and aging anxiety among pre-elderly individuals in China and the Republic of Korea and obtained several insightful results. First, the levels of PA, ageism, and aging anxiety differed significantly between Chinese and Korean pre-elderly individuals. The PA level was higher among pre-elderly Korean individuals than among their Chinese counterparts.

PA levels vary substantially between countries and regions, and the phenomenon of insufficient PA is increasing worldwide owing to various factors, including regional public services, economic conditions, sex, and age [33]. The study has shown that this phenomenon is especially prevalent in five countries, namely, the USA, Japan, Canada, Australia, and the UK, where absolute and relative declines in total PA are the highest and sedentary time is also high [34]. Ding et al. [35] found that PA levels among older Chinese people are lower than the World Health Organization’s recommended level and the levels among people from some developed countries. A follow-up survey conducted in China between 2011 and 2012 revealed that the rate of insufficient PA among individuals aged 45 years and older was 44.06%. These results may reflect differences in public health messaging or cultural attitudes toward exercise, as discussed by Li et al. [36]. Gu et al. [37] reported that economic conditions and living areas (urban or rural) had a direct impact on public sports activities at all intensity levels. Older individuals living in economically developed areas reported higher levels of moderate and moderate to vigorous PA. These results confirm the findings of the present study. There was a gap in the economic conditions, living areas, consumption levels, and welfare systems of the two countries examined in this study, which may have led to lower levels of PA among Chinese participants than among Korean participants. Li et al. [36] found that the lack of knowledge about PA and fitness, as well as limited guidance and activity content, are key factors affecting the participation of middle-aged and older individuals in PA. These conditions stem from inefficient public service systems. Therefore, government agencies should modernize public service systems and provide facilities for PA to support middle-aged and older adults.

Second, levels of ageism and aging anxiety were higher among pre-elderly Chinese individuals than among their Korean counterparts. Ageism can be expressed consciously or subconsciously at the micro (individual), meso (social network), and macro (institutional and cultural) levels [38]. Studies have shown that the prevalence of ageism varies greatly between countries, and such instances are influenced by cultural, socioeconomic, and political factors [39] rather than stemming from physiological or maturity differences between people of different ages [40]. Officer et al. [41] showed that the likelihood of age discrimination is five times higher in low- and middle-income countries than in high-income countries. The income of middle-aged and older individuals differs between China and the Republic of Korea, which may help explain the higher levels of ageism in China compared with those in the Republic of Korea. Studies have also reported that individuals with higher educational levels are less likely to have negative perceptions of older people [42]. Other cross-cultural studies have found that individuals with higher levels of education tend to believe that old age begins later and do not identify themselves as old, thereby protecting themselves from self-directed age discrimination [43]. In this study, the proportion of Chinese participants with a college degree or higher was 31.5%, which was significantly lower than the proportion among Korean participants (76.1%). The understanding of age discrimination across different cultures remains nascent [44]. Comparisons between a few countries may not accurately reflect cultural differences; however, they can offer valuable insights into aspects of culture that are not yet fully understood.

Age categorization is socially and psychologically problematic and can lead people to define what is acceptable for a particular age group based on their own or others’ ageist assumptions. For example, people may judge or believe that they are “too young” or “too old” to pursue a particular activity or role. Therefore, the categorization of people into age groups and the way people define these groups have a significant impact on people’s attitudes and behaviors [45].

Third, PA had no effect on ageism among pre-elderly individuals. Previous studies have shown that PA levels can be influenced by psychological factors, including personality traits, social comparisons, and even stereotypes [46]. In a cross-sectional study, Emile et al. [47] found that the more pessimistic the stereotypes that older individuals believe about their bodies, the lower their PA levels. Regular PA has a positive impact on the health of older individuals, and PA for a particular duration is associated with a reduced risk of chronic disease. However, the results of this study showed no impact of PA. Further, studies have shown that the prevalence of ageism varies greatly between countries and is influenced by cultural, socioeconomic, and political factors [39,48,49]. Differences in ageism may also occur due to cultural factors that prioritize group norms over individual behavior. Thus, follow-up studies are warranted to clarify these aspects.

Fourth, PA had a significantly negative effect on the physical weakness aspect of aging anxiety, indicating that physical exercise can relieve anxiety in pre-elderly individuals regarding the deterioration of physical function. This result is consistent with that of Wanjau et al. [50], who found that regular participation in PA reduced anxiety and depression levels among older individuals. Similarly, Oh et al. [51] reported that PA has a negative impact on aging anxiety and that higher levels of PA are related to better health. The effects of aging, such as functional decline in muscle mass, speed, strength, stability, and elasticity, are associated with outcomes such as vulnerability and disease and contribute to limiting overall well-being [52]. However, PA can effectively counteract these effects. Furthermore, many studies have demonstrated that regular PA can effectively reduce cardiovascular disease [53] and help prevent depression [54,55], dementia [56], and anxiety [57,58]. In a social context, PA promotes social integration and provides older adults with activities that contribute to an active lifestyle, such as recreational sports, cultural activities, dance classes, intellectually stimulating games, craft activities, and group interactions. These activities enable middle-aged and older adults to engage in a greater level of social participation as they enter an older age.

Finally, differential treatment and stereotypes had a significantly positive effect on the social worthlessness and appearance anxiety aspects of aging. Ageism can manifest in two ways: it can be directed toward others (other-oriented ageism) or oneself (self-oriented ageism) [59]. Related research has shown that age discrimination against oneself or others largely stems from internalized age stereotypes. People tend to internalize the negative perceptions of older individuals, and these perceptions shape their perceptions of aging as they grow up [60,61]. As people age, they feel increasingly useless and believe that aging leads to many negative consequences [61]. In fact, even older individuals often harbor negative perceptions of aging and other older people, which may partly explain why aging and the changes associated with it are frequently feared and avoided rather than celebrated [62]. A longitudinal study by Becca et al. [63] found that at the individual level, individuals with a positive attitude toward aging live 7.5 years longer than those with a negative attitude.

Molden and Maxfield [64] investigated the impact of attitudes toward aging on concerns about dementia among older adults. They used an intervention to expose older individuals to positive and negative aging stereotypes. They found that those exposed only to negative stereotypes exhibited higher levels of dementia concerns. This result highlights the significant impact of attitudes toward aging on the lives of older adults. Similar results were obtained by Ishikawa [65] and Kahraman et al. [66], who found that ageism is positively related to death anxiety. Allan and Johnson [67] showed that endorsing ageist beliefs and attitudes is associated with greater aging anxiety; that is, the more anxious a person is about getting older, the more they tend to have negative prejudices against older people. Meanwhile, a recent study using longitudinal data from the Health and Retirement Study [68] found that positive perceptions of aging can enhance physical recovery and social reengagement among older individuals after a fall.

This study had some limitations. We did not assess participants’ cognitive ability prior to the administration of the questionnaire. For studies involving psychological aspects, it is advisable to assess cognitive ability/chronic conditions prior to the study. Further, in this study, we relied on self-reported data on PA levels and measures of perceived aging. This approach, especially in middle-aged and older adults, is associated with inevitable biases, such as participants’ overestimation of PA and recall bias. In addition, since this research was conducted in a single region of China and Korea, the results may not be generalizable as being representative of the entire country. Furthermore, the participants’ nutritional status, PA level, and smoking, drinking, drug, and other lifestyle habits were not investigated. Nonetheless, this study can serve as a good reference for future international comparative studies as it provides data comparing the two countries of China and Korea.

## 5. Conclusions

Our findings indicate that PA may have an effect on ageism and aging anxiety among pre-elderly individuals in China and the Republic of Korea. Therefore, awareness of the importance of PA should be increased among pre-elderly Chinese and Korean individuals, and efforts should be made to encourage them to engage in more PA. It is necessary to implement social policies that reduce ageism and aging anxiety, respect autonomy and personal control, involve older people in the design of healthy aging strategies, and enhance awareness of healthy aging. For instance, the government can work with the media to launch a national “active aging” campaign or set up healthy aging service stations in the community, ensuring that pre-elderly individuals are adequately prepared for healthy aging. Furthermore, more research must be conducted on age discrimination to better understand its roots in our culture, its manifestations, and its impact on the lives and health of older adults. This area of research remains underexplored in China and Korea, and more data and research measures are required to study age discrimination.

## Figures and Tables

**Table 1:** General characteristics of the participants.

Variable		Chinese *n* (%)	Korean *n* (%)	Total *n* (%)
Sex	Men	72 (36.00)	59 (29.35)	131 (32.67)
	Women	128 (64.00)	142 (70.65)	270 (67.33)
Age	55–59	154 (77.00)	115 (57.21)	269 (67.08)
	60–64	46 (23.00)	86 (42.79)	132 (32.92)
Body mass index (kg/m^2^)	Underweight (<18.5)	6 (3.00)	5 (2.48)	11 (2.74)
	Normal (18.5–22.9)	68 (34.00)	109 (54.23)	177 (44.14)
	Overweight (23.0–24.9)	55 (27.50)	46 (22.89)	101 (25.18)
	Preobesity (25.0–29.9)	64 (32.00)	40 (19.90)	104 (25.94)
	Obese (≥30.0)	7 (3.50)	1 (0.50)	8 (2.00)
Marital status	Unmarried	3 (1.50)	9 (4.47)	12 (2.99)
	Married	192 (96.00)	182 (90.55)	374 (93.26)
	Divorced	2 (1.00)	6 (2.99)	8 (2.00)
	Widowed	3 (1.50)	4 (1.99)	7 (1.75)
Smoking status	Smoking	34 (17.00)	18 (8.96)	52 (12.97)
	Nonsmoking	166 (83.00)	183 (91.04)	349 (87.03)
Education level	Primary school	14 (7.00)	0 (0.00)	14 (3.49)
	Junior high school	123 (61.50)	48 (23.88)	171 (42.64)
	University graduate	55 (27.50)	113 (56.22)	168 (41.90)
	Graduate and above	8 (4.00)	40 (19.90)	48 (11.97)
Total		200	201	401

**Table 2:** Results of testing reliability and validity of the scales employed.

Variable		Item	Estimate	Squared Multiple Correlation	Standardized Residuals	Composite Reliability	Average Variance Extracted	Cronbach α	
Ageism	Stereotype	16	0.763	0.582	0.418	0.834	0.421	0.794	0.850
		18	0.716	0.513	0.487				
		13	0.652	0.425	0.575				
		15	0.650	0.423	0.578				
		17	0.632	0.399	0.601				
		14	0.569	0.324	0.676				
		9	0.529	0.280	0.720				
	Discrimination attitude	7	0.829	0.687	0.313	0.828	0.548	0.796	
		10	0.747	0.558	0.442				
		5	0.731	0.534	0.466				
		8	0.642	0.412	0.588				
	Emotional avoidance	1	0.796	0.634	0.366	0.836	0.629	0.795	
		3	0.792	0.627	0.373				
		2	0.792	0.627	0.373				
Kaiser–Meyer–Olkin = 0.857, χ^2^ = 1934.487, df = 91 (*p* < 0.001)									
Aging anxiety	Social worthlessness	12	0.825	0.681	0.319	0.897	0.525	0.903	0.899
		13	0.798	0.637	0.363				
		14	0.791	0.626	0.374				
		11	0.763	0.582	0.418				
		10	0.722	0.521	0.479				
		9	0.691	0.477	0.523				
		15	0.634	0.402	0.598				
		16	0.521	0.271	0.729				
	Physical weakness	1	0.835	0.697	0.303	0.842	0.576	0.832	
		2	0.818	0.669	0.331				
		3	0.778	0.605	0.395				
		4	0.576	0.332	0.668				
	Appearance anxiety	5	0.774	0.559	0.401	0.781	0.474	0.817	
		6	0.706	0.498	0.502				
		7	0.694	0.482	0.518				
		8	0.563	0.317	0.683				
	Expectations of old age	19	0.849	0.721	0.279	0.878	0.706	0.807	
		17	0.840	0.706	0.294				
		18	0.832	0.692	0.308				
Kaiser–Meyer–Olkin = 0.913, χ^2^ = 4112.611, df = 171 (*p* < 0.001)									

**Table 3:** Results of comparing PA, ageism, and aging anxiety among the participants based on nationality, sex, body mass index, marital status, smoking status, and education level.

Characteristic		Categories				
		Total PA			Ageism	Aging Anxiety
		High PA	Moderate PA	Low PA		
Nationality	Chinese (*n* = 200)	6067.45 ± 2942.07	1543.27 ± 680.75	343.29 ± 171.54	3.05 ± 0.57	3.16 ± 0.55
	Korean (*n* = 201)	5915.59 ± 2628.94	1764.25 ± 707.14	394.89 ± 144.85	2.74 ± 0.56	2.97 ± 0.59
t (*p*)		−2.221 (0.027 *)			5.368 (<0.001 ***)	3.312 (0.001 **)
Sex	Men	6177.41 ± 3021.07	1645.64 ± 747.07	327.20 ± 185.65	2.89 ± 0.61	3.09 ± 0.62
	Women	5842.42 ± 2567.41	1657.64 ± 685.62	381.03 ± 149.27	2.90 ± 0.57	3.06 ± 0.56
t (*p*)		2.271 (0.024 *)			−0.138 (0.890)	0.550 (0.583)
Body mass index (kg/m^2^)	Underweight (<18.5)	1941.82 ± 2012.15	1014.55 ± 1027.19	2718.00 ± 1720.07	3.04 ± 0.48	3.25 ± 0.54
	Normal (18.5–22.9)	1060.79 ± 1632.89	712.77 ± 883.88	1141.87 ± 976.21	2.83 ± 0.61	3.01 ± 0.61
	Overweight (23.0–24.9)	1159.60 ± 1816.64	777.82 ± 1133.12	1276.71 ± 1204.79	2.84 ± 0.57	3.05 ± 0.47
	Preobesity (25.0–29.9)	754.62 ± 1486.45	388.46 ± 765.66	1053.62 ± 1035.95	2.99 ± 0.53	3.11 ± 0.59
	Obese (≥30.0)	240.00 ± 339.41	125.00 ± 157.75	1018.88 ± 985.34	3.10 ± 0.58	3.51 ± 0.40
F (*p*)		2.205 (0.068)	3.848 (0.004 **)	6.247 (<0.001 ***)	1.872 (0.115)	2.100 (0.080)
Marital status	Unmarried	5433.00 ± 2239.51	1587.88 ± 677.76	396.00 ± 0.00	2.57 ± 0.53	2.66 ± 0.62
	Married	5899.47 ± 2779.11	1655.27 ± 7.3.46	362.76 ± 163.71	2.91 ± 0.59	3.08 ± 0.68
	Divorced	7518.00 ± 3134.91	1642.50 ± 786.56	-	2.62 ± 0.39	3.13 ± 0.54
	Widowed	7679.50 ± 2159.91	1714.50 ± 1024.59	-	3.06 ± 0.39	3.05 ± 0.23
F (*p*)		1.966 (0.118)	3.993 (0.008 **)	0.410 (0.746)	2.087 (0.101)	2.108 (0.099)
Smoking status	Smoking	5567.78 ± 2093.30	1324.11 ± 672.52	267.38 ± 197.08	2.98 ± 0.65	3.08 ± 0.61
	Nonsmoking	6068.40 ± 2879.34	1687.66 ± 697.13	383.71 ± 148.78	2.88 ± 0.57	3.07 ± 0.57
t (*p*)		0.384 (0.701)			1.137 (0.256)	0.233 (0.816)
Education level	Primary school	5886.00 ± 3925.51	1596.00 ± 676.26	263.00 ± 196.50	2.95 ± 0.57	3.27 ± 0.48
	Junior high school	5910.27 ± 2792.80	1564.28 ± 688.92	365.84 ± 160.47	2.95 ± 0.60	3.18 ± 0.56
	University graduate	5991.75 ± 2862.32	1761.28 ± 700.11	389.15 ± 157.93	2.85 ± 0.55	2.99 ± 0.58
	Graduate and above	6230.62 ± 1892.15	1650.00 ± 741.66	299.25 ± 187.61	2.80 ± 0.64	2.93 ± 0.57
F (*p*)		1.110 (0.345)			1.342 (0.260)	4.833 (0.003 **)

Data are expressed as mean ± standard deviation; PA, physical activity, * *p* < 0.05, ** *p* < 0.01, *** *p* < 0.001, tested using independent *t*-test and one-way analysis of variance.

**Table 4:** Results of testing the suitability of the research model.

Model	Χ^2^	df	GFI	IFI	TLI	CFI	RMSEA
Model fit	129.855	64	0.943	0.938	0.910	0.936	0.051

GFI: goodness-of-fit index; IFI: incremental fit index; TLI: Tucker–Lewis index; CFI: comparative fit index; RMSEA: root mean square error of approximation. Model fit cutoff values: RMSEA < 0.100, TLI ≥ 0.900, CFI ≥ 0.900

**Table 5:** Results of testing path relationships among PA, ageism, and aging anxiety.

Hypothesis			Path			β	Standard Error	Critical Ratio	*p*	Assessment
H1	Physical activity		→	Ageism	Emotional avoidance	−0.058	0.055	−1.060	0.298	Reject
			→		Discrimination attitude	−0.061	0.039	−1.564	0.118	Reject
			→		Stereotype	−0.011	0.050	−0.215	0.830	Reject
H2	Physical activity		→	Aging anxiety	Social worthlessness	0.001	0.045	0.025	0.981	Reject
			→		Physical weakness	−0.164	0.058	−2.585	0.044 *	Accept
			→		Appearance anxiety	0.016	0.037	0.428	0.669	Reject
			→		Expectations of old age	−0.022	0.051	−0.441	0.660	Reject
H3	Ageism	Emotional avoidance	→	Aging anxiety	Social worthlessness	−0.036	0.051	−0.709	0.478	Reject
			→		Physical weakness	0.059	0.045	1.315	0.188	Reject
			→		Appearance anxiety	0.028	0.042	0.660	0.509	Reject
			→		Expectations of old age	0.042	0.058	0.724	0.469	Reject
	Ageism	Discrimination attitude	→	Aging anxiety	Social worthlessness	0.260	0.071	3.651	<0.001 ***	Accept
			→		Physical weakness	−0.013	0.059	−0.215	0.829	Reject
			→		Appearance anxiety	0.182	0.059	3.095	0.002 **	Accept
			→		Expectations of old age	−0.165	0.078	−2.120	0.034 *	Accept
	Ageism	Stereotype	→	Aging anxiety	Social worthlessness	0.448	0.066	6.737	<0.001 ***	Accept
			→		Physical weakness	0.527	0.065	8.131	<0.001 ***	Accept
			→		Appearance anxiety	0.459	0.064	7.219	<0.001 ***	Accept
			→		Expectations of old age	−0.102	0.064	−1.589	0.112	Reject

* *p* < 0.05, ** *p* < 0.01, *** *p* < 0.001; tested using path analysis.

## Data Availability

The data presented in this study are available upon request from the corresponding author. Data were not publicly available to protect personal information.
